# Angiotensin II, blood–brain barrier permeability, and microglia interplay during the transition from pre-to hypertensive phase in spontaneously hypertensive rats

**DOI:** 10.3389/fphys.2024.1452959

**Published:** 2024-09-12

**Authors:** Mariana Makuch-Martins, Camilla G. Vieira-Morais, Sany M. Perego, Adriana Ruggeri, Alexandre Ceroni, Lisete C. Michelini

**Affiliations:** Department of Physiology and Biophysics, Institute of Biomedical Sciences, University of Sao Paulo, São Paulo, Brazil

**Keywords:** angiotensin II, blood–brain barrier, microglia, autonomic control, spontaneously hypertensive rats

## Abstract

**Background:**

Hypertension is characterized by upregulation of the renin–angiotensin system, increased blood–brain barrier (BBB) permeability, microglia activation within autonomic nuclei, and an intense sympathoexcitation. There is no information on the interplay of these events during the development of neurogenic hypertension. We sought to identify the interaction and time-course changes of Ang II availability, barrier dysfunction, microglia activation, and autonomic imbalance within autonomic areas during the development of neurogenic hypertension.

**Methods:**

Sequential changes of hemodynamic/autonomic parameters, BBB permeability, microglia structure/density (IBA-1), and angiotensin II **(**Ang II) immunofluorescence were evaluated within the paraventricular hypothalamic nucleus, nucleus of the solitary tract, and rostral ventrolateral medulla of Wistar and spontaneously hypertensive rats (SHRs) aged 4 weeks, 5 weeks, 6 weeks, 8 weeks, and 12 weeks. The somatosensory cortex and hypoglossal nucleus were also analyzed as non-autonomic control areas.

**Results:**

Increased brain Ang II availability (4th–5th week) was the first observed change, followed by the incipient BBB leakage and increased microglia density (6th week). From the 5th–6th weeks on, BBB leakage, Ang II, and IBA-1 densities increased continuously, allowing a parallel increase in both Ang II-microglia colocalization and the transition of microglial cells from highly ramified in the basal surveillant condition (4th–5th week) to shorter process arbors, fewer endpoints, and enlarged soma in the disease-associate condition (6th week to the 12th week). Simultaneously with increased Ang II-microglia colocalization and microglia morphologic phenotypic changes, sympathetic activity and pressure variability increased, autonomic control deteriorated, and blood pressure increased. These responses were not specific for autonomic nuclei but also occurred at a lower magnitude in the somatosensory cortex and hypoglossal nucleus, indicating the predominance of hypertension-induced effects on autonomic areas. No changes were observed in age-matched controls where Ang II density did not change.

**Conclusion:**

Brain Ang II density is the initial stimulus to drive coordinated changes in BBB permeability and microglial reactivity. Increased BBB dysfunction allows access of plasma Ang II and increases its local availability and the colocalization and activation of microglial cells. It is a potent stimulus to augments vasomotor sympathetic activity, autonomic imbalance, and pressure elevation during the establishment of hypertension.

## Introduction

It is well known that hypertension, a low-grade inflammatory disease, is accompanied by upregulation of the renin–angiotensin system, dysfunctional blood–brain barrier (BBB) permeability, and resident microglia activation within autonomic brain areas, allowing the autonomic imbalance that maintains high vasomotor sympathetic activity and pressure elevation (Guyenet, 2006; Biancardi et al., 2014; Shen et al., 2015; Buttler et al., 2017; Forrester et al., 2018; Su et al., 2021; Wang et al., 2022).

The BBB, a complex multicellular structure laying on the basement membrane, is composed of endothelial cells linked by tight junction protein complexes, which are enveloped by pericytes and astrocyte endfeet (Abbott et al., 2006). Conflicting observations are reported for dysfunctional BBBs in hypertensive animals, including tight junction loss/breakdown (Pelisch et al., 2013; Mohammadi and Dehghani, 2014; Zhang et al., 2015) as well as intact tight junctions with dysfunctional transcellular transport (Ueno et al., 2004; Fragas et al., 2021; Candido et al., 2023). Microglia are not structural components of the BBB but release several soluble factors that can alter the barrier function (Abbott et al., 2006; Keaney and Campbell, 2015). It should be noted that microglia are extremely dynamic cells continuously surveying the brain parenchyma under both physiological and pathological conditions and thus participate in several critical brain functions (Paolicelli et al., 2022).

Although microglial research has advanced considerably for many neurodegenerative conditions such as Alzheimer’s disease, multiple sclerosis, amyotrophic lateral sclerosis, Parkinson's disease, and aging (Paolicelli et al., 2022), less information on microglial state/function is available in hypertension. By using an intracerebroventricular infusion of an anti-inflammatory antibiotic, Shi et al. (2010) demonstrated that Ang II-induced hypertension involves the activation of microglial cells and the increase of pro-inflammatory cytokines in the paraventricular nucleus of the hypothalamus (PVN). It was also shown that both blockade of microglial activation and its targeted depletion within the PVN attenuate microglia expression and the synthesis of pro-inflammatory cytokines, decrease sympathetic activity, and reduce blood pressure in both Ang II- and high salt-induced hypertension (Shen et al., 2015; Yu et al., 2022). Microglia inflammatory mediators are shown to be important effectors to disrupt the BBB, increasing barrier permeability and causing autonomic imbalance in hypertension (Setiadi et al., 2018).

Some years ago, Biancardi et al. (2014) reported an intense BBB disruption within autonomic areas with extravasation of plasma Ang II into the brain parenchyma colocalizing with neurons and microglial cells in 13-week-old SHRs. Importantly, we showed that the increased BBB permeability in autonomic nuclei was positively correlated with robust autonomic dysfunction and high vasomotor sympathetic activity in 3-month-old SHRs (Buttler et al., 2017). More recent studies indicated that the absorptive transcytosis across the endothelial cell (not changes in the paracellular transport) was the key mechanism for increased barrier permeability (Fragas et al., 2021; Candido et al., 2023). Indeed, the increased vesicular transport by allowing the entrance of plasma Ang II into the brain parenchyma increased the hormone availability within autonomic nuclei and potentiated the activation of pre-sympathetic neurons. Mowry et al. (2021) also reported that elevated brain Ang II availability activated the microglial cells, augmented the synthesis of pro-inflammatory cytokines and increased BBB leakage because its blockade normalized the high barrier permeability exhibited by the SHR. These studies in conscious SHRs were focused only on the chronic phase of hypertension and did not evaluate the intrinsic interaction between Ang II availability, microglia activation, and BBB dysfunction. It is our working hypothesis that an intense interplay between Ang II availability, BBB permeability, and microglia activation within autonomic nuclei continuously drives the sympathoexcitation and the autonomic dysfunction during the transition from pre-to the hypertensive phase.

Therefore, in the present observational study, we evaluated the time-course changes of hemodynamic/autonomic parameters, BBB permeability, microglia density, and their morphological changes, as well as Ang II availability in important autonomic nuclei, including the PVN, the nucleus of the solitary tract (NTS), and the rostral ventrolateral medulla (RVLM), during the establishment of hypertension in SHRs. Age-matched normotensive rats were used as controls. We also analyzed the sequential changes within the primary somatosensory cortex (C_SS_) and the hypoglossal nucleus (12N) in the same rats to uncover whether changes were or were not specific to autonomic areas.

## Methods

### Ethical approval

All surgical procedures and experimental protocols were reviewed and approved by the Institutional Animal Care and Use Committee of the University of Sao Paulo (CEUA, protocol no. 3112251119) in compliance with the Ethical Principles in Animal Research of the Brazilian College in Animal Experimentation.

### Animals and experimental design

Male 3-week-old spontaneously hypertensive rats (SHRs) and age-matched Wistar controls were housed on a 12–12-h light–dark cycle with free access to standard chow and water in the Animal Facilities of the Department of Physiology & Biophysics, University of Sao Paulo. Hemodynamic/autonomic recordings, analysis of BBB permeability, and evaluation of both Ang II and microglia immunofluorescence were performed in SHRs and Wistar rats aged 4 weeks, 5 weeks, 6 weeks, 8 weeks, and 12 weeks. One day before the pre-determined times, the rats were pre-anesthetized with acepromazine (2.5 mg/kg *sc*), followed by isoflurane (5% induction; 2% maintenance) for catheterization of the femoral artery. They were treated subcutaneously with meloxicam (1 mg/kg, *sc*.) and enrofloxacin (10 mg/kg, *sc*.) and returned to their home cages for recovery.

### Hemodynamic and autonomic measurements

Resting pulsatile arterial pressure (AP) and heart rate (HR) were continuously recorded on a beat-to-beat basis (50–60 min, LabChart Pro, ADInstruments, sampling frequency of 2,000 Hz) in conscious unrestrained rats resting in their home cages (Ichige et al., 2016; Candido et al., 2023). Time series of systolic AP (SAP) and pulse interval (PI) were used to evaluate SAP and HR variabilities at the frequency domain (Buttler et al., 2017; Fragas et al., 2021). Power spectral density for the low frequency (LF, 0.20–0.75 Hz, indicative of sympathetic vasomotor activity and sympathetic + parasympathetic activity to the heart), the high frequency(HF, >0.75–3.00 Hz, indicative of cardiac vagal modulation), and the spontaneous baroreflex sensitivity (αHF) were also evaluated as previously described (Masson et al., 2014).

### Analysis of BBB permeability

After functional recordings at the 4th, 5th, 6th, 8th, and 12th weeks, some of the SHRs and Wistar rats of each group were anesthetized (acepromazine 2.5 mg/kg followed by ketamine, 100 mg/kg + xylazine 20 mg/kg *i. p.*) for catheterization of the right carotid artery as previously described (Buttler et al., 2017; Fragas et al., 2021; Candido et al., 2023; Perego et al., 2023). Briefly, a mixture of fluorescent dyes of different molecular sizes—fluorescein isothiocyanate dextran 10 kDa (FITC, 10 mg/mL, Sigma-Aldrich) and rhodamine isothiocyanate dextran 70 kDa (RHO, 10 mg/mL, Sigma-Aldrich), 286 mL/100 g each—was centrally administered into the brain vasculature at a slow rate and allowed to circulate for 30 min. Rats were then euthanized (overdose of anesthesia) for brain harvesting immediately after the respiratory arrest. Brains were then fixed (4% phosphate-buffered paraformaldehyde, 48 h), cryoprotected (20% and 30% sucrose in 0.01 M PBS, 3 days at 4°C), and stored until processing. Sequential coronal PVN, NTS, and RVLM slices (30 μm, Leica CM1850 cryostat, Nussloch, Germany) were collected and mounted in gelatinized slides. The C_SS_ and 12N were also analyzed in the same slices as non-autonomic control areas. Images were examined in specific ROIs by a blind observer on a fluorescence microscope (Leica DMLB) attached to an ExiBlue camera (Imaging, Canada), acquired (Image-Pro Plus software, Media Cybernetics), and analyzed by the ImageJ software (NIH, United States) according to the technique previously described by Biancardi et al. (2014). BBB permeability was evaluated by the capability of small-size FITC-10kDa to remain within the intact microvasculature (capillaries and a few small venules) or partially leak into the brain parenchyma in the presence of compromised barrier integrity. The large rhodamine 70 kDa should be contained within the microvasculature and not leak even in the presence of altered BBB permeability. BBB leakage was quantified bilaterally within the specific ROIs in all areas analyzed using 6–8 slices/rat and values averaged to yield a single value/area/rat.

### Immunofluorescence assays

Other SHRs and Wistar rats aged 4 weeks, 5 weeks, 6 weeks, 8 weeks, and 12 weeks were euthanized by an overdose of anesthetics after the functional recordings and perfused immediately after the respiratory arrest with Dulbecco’s modified Eagle’s medium (D-8900, Sigma-Aldrich, MS, United States; Daigger Pump, IL, United States), followed by the fixative (4% PFA in 0.1 M PBS), as previously described (Rocha-Santos et al., 2020). Briefly, brains were removed, postfixed (4% PFA 0.1 M for 24 h), cryoprotected (0.1 M Tris-PBS containing sucrose for 48 h), and stored at 4°C until processing. Coronal sections of the PVN, NTS, RVLM, C_SS_, and 12N (Paxinos and Watson, 2013) were collected and processed as previously described (Fragas et al., 2021; Perego et al., 2023). Slices were pretreated with 1% sodium borohydride, immersed in a solution containing 1% hydrogen peroxide and 10% methanol in 0.1 M PBS, washed with KPBS 0.02 M, and blocked with 0.3% Triton X-100 in 2% donkey serum. Sections suspended in 0.3% Triton X-100 in 2% donkey serum were incubated for 24 h with a mixture of primary antibodies: polyclonal rabbit anti-ionized calcium-binding adapter molecule 1 (IBA-1, 1:1000 dilution, FujiFilm Wako Chemicals, Osaka Japan, Cat. No.019–19741), a specific microglial marker raised against the C-terminus of IBA-1 that does not cross-react with neurons and astrocytes, and polyclonal guinea pig anti-angiotensin II (T-5001, 1:250 dilution, BMA Biomedicals/Peninsula Laboratories, Switzerland), an antibody generated by immunization with Ang II (100% cross-reaction with Ang II) that has been previously tested and validated by ELISA, which shows very little cross-reaction with Ang I (0.8%) and AGT (0.3%). Tissues were then washed and subjected to 1-h incubation (0.02 M KPBS, 0.3% Triton X-100, 1% donkey serum) at room temperature with secondary antibodies: anti-rabbit Alexa Fluor 488 (Cat. No. 711–545–152) and anti-guinea pig Alexa Fluor 594 (Cat. No. 706–585–148), 1:500 dilution each, Jackson ImmunoResearch MD, United States). After three 10-min washes in 0.02 M KPBS, tissues were mounted in gelatinized slides and cover slip with slowfade (Prolong™ Gold Antifade Mount, CA, United States) and stored in the dark at 4°C.

PVN, NTS, RVLM, C_SS_, and 12N sections were carefully examined in a fluorescence microscope (Leica DMLB) by a blind investigator. IBA-1 and Ang II immunofluorescence signals were acquired in the same ROIs used for BBB analysis. Images were analyzed with identical acquisition settings. The calculation of background fluorescence, the threshold setting, and the quantification of the immunoreactivity were made as previously described (Rocha-Santos et al., 2020). Image analysis was performed with ImageJ software (NIH) and expressed as the integrated density of the thresholded signal/ROI (arbitrary units). Negative controls omitted the primary antibodies. In the double-labeled slides, we also quantified the association of Ang II with microglial cells using their binary images and the command “colocalization” of the ImageJ. Measurements were made in both the left and right sides of the nuclei, 6–8 slices/rat; values were averaged to give a mean value for the nucleus for each rat in each condition.

### Quantification of microglia morphology

The effects of establishing hypertension on microglial morphology were automatically quantified by NeurphologyJ (Ho et al., 2011), a freely available plugin to ImageJ. NeurphologyJ automatically analyzed the microglial cell number, their processes number and length and end points into the PVN, NTS, RVLM, C_SS_, and 12N within each ROI. In addition, the ratio between the total pixel area within cell bodies and the number of microglial cells allowed us to calculate the soma size index.

### Statistical analysis

Data were expressed as mean ± SEM. All results were submitted to the homogeneity variance test (Shapiro–Wilk). Functional measurements, BBB permeability, Ang II, and IBA-1 densities, their colocalization, and microglial morphology between ages and groups were analyzed by two-way factorial ANOVA or one-way ANOVA as appropriate. Tukey’s test was the *post-hoc* test. Analyses were performed using the GraphPad^®^ Prism 8 software (San Diego, CA, United States). Differences were considered significant at *p* < 0.05.

## Results

### Sequential changes on hemodynamic and autonomic recordings

**FIGURE 1:**
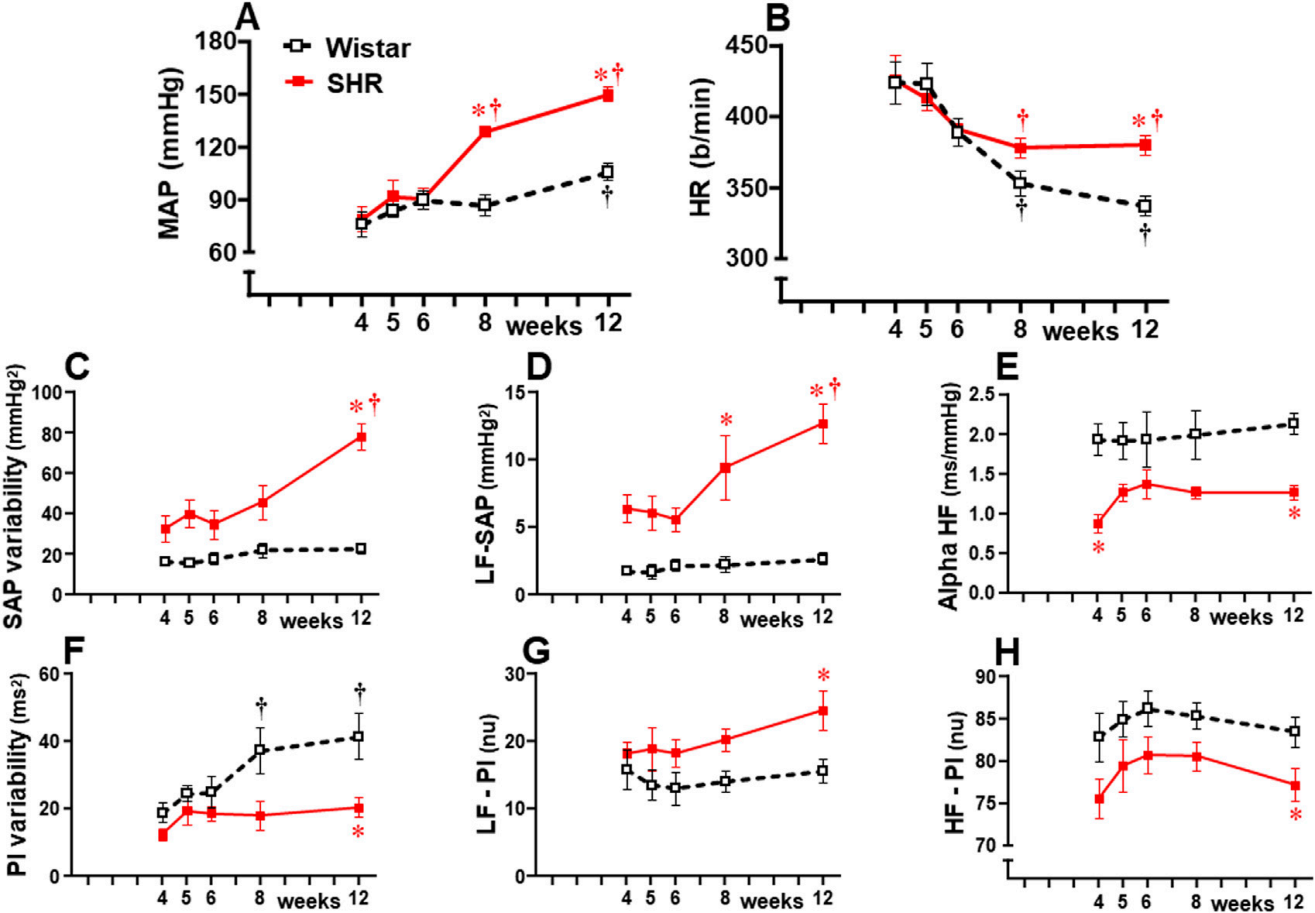
Hemodynamic and autonomic recordings in SHRs and Wistar rats from 4 weeks to 12 weeks of age. Baseline mean arterial pressure [MAP, **(A)]** and heart rate [HR, **(B)]**, systolic arterial pressure [SAP, **(C)]**, and pulse interval [PI, **(F)]** variabilities with their spectral components at low [LF-SAP, **(D)** and LF-PI, **(G)]** and high frequencies [HF-PI, **(H)]**, and spontaneous baroreflex sensitivity (alpha HF, **(E)**. *n =* 6–9 rats/group. Comparisons made by two-way factorial ANOVA. MAP: *group* F (1,61) = 26.18 *P* < 0.001, *age* F (4,61) = 23.33 *P* < 0.001, *interaction* F (4,61) = 6.45, *P* = 0.001; HR: *group* F (1,61) = 3.78 *P* = 0.050, *age* F (4,61) = 13.88 *P* < 0.001, *interaction* F (4,61) = 1.68 *P* = 0.166; SAP variability: *group* F (1,61) = 57.61 *P* < 0.001, *age* F (4,61) = 8.67 *P* < 0.001, *interaction* F (4,61) = 5.16 *P* = 0.001; PI variability: *group* F (1,61) = 19.10 *P* < 0.001, *age* F (4,61) = 4.79 *P* = 0.002, *interaction* F (4,61) = 1.80 *P* = 0.136; LF-SAP: *group* F (1,61) = 67.96 *P* < 0.001, *age* F (4,61) = 5.17 *P* = 0.001, *interaction* F (4,61) = 3.36 *P* = 0.014; LF-PI: *group* F (1,61) = 11.72 *P* = 0.001, *age* F (4,61) = 1.08 *P* = 0.373, *interaction* F (4,61) = 0.53 *P* = 0.717; HF-PI: *group* F (1,61) = 15.54 *P* < 0.001, *age* F (4,61) = 1.30 *P* = 0.277, *interaction* F (4,61) = 0.08 *P* = 0.987; Alpha HF: *group* F (1,61) = 38.43 *P* < 0.001, *age* F (4,61) = 0.82 *P* = 0.516, *interaction* F (4,61) = 0.55 *P* = 0.701; Significances (*P* < 0.05): * vs. age-matched Wistar; † vs. respective week 4.

Hemodynamic recordings from the pre-hypertensive up to the chronic phase of hypertension showed that mean AP (MAP) and HR values were similar in both strains at 4 weeks, 5 weeks, and 6 weeks of age (Figures 1A, B). From 8 weeks to 12 weeks, SHRs exhibited pronounced MAP increases, while age-matched Wistar rats showed no change or a very mild increase at the end of the protocol. In both groups, baseline HR values, highly elevated at the pre-hypertensive phase, exhibited significant reduction by the 8th week, with no further change in the SHRs, while Wistar rats showed a progressive decrease to the end of the experimental protocol (Figure 1B). Analysis of the autonomic parameters revealed that pressure elevation in SHRs occurred simultaneously with pronounced increases in vasomotor and cardiac sympathetic activities (Figures 1D, G, respectively) and a reduction of parasympathetic activity of the heart (Figure 1H). These changes caused a continuous increase in pressure variability (Figure 1C) and blocked the progressive increase in heart rate variability exhibited by Wistar rats (Figure 1F). Autonomic imbalance in the SHR group was also characterized by reduced spontaneous baroreflex sensitivity (Figure 1E). Except for increased heart rate variability, normotensive rats showed no significant changes in autonomic parameters from 4 weeks to 12 weeks of age.

### Sequential changes on BBB function and microglia density within autonomic areas

**FIGURE 2:**
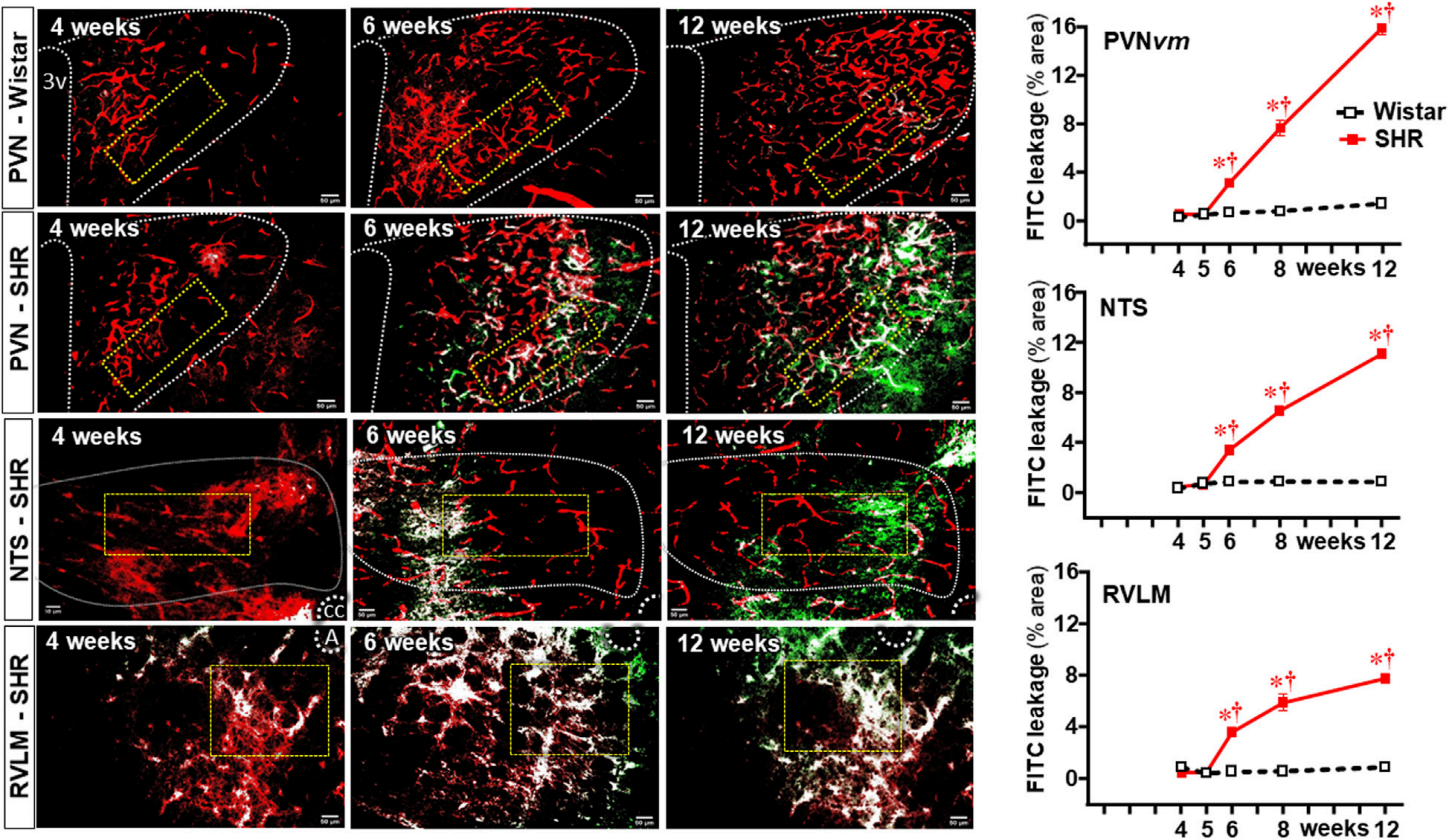
BBB permeability changes within autonomic nuclei during the development of hypertension. Representative images of SHRs (PVN, NTS, and RVLM) and Wistar rats (PVN) aged 4 weeks, 6 weeks, and 12 weeks illustrate the capillary profile (rhodamine-70kDa, red), the FITC-10 kDa leakage into the brain parenchyma (green) and the colocalization of both (white). The superimposed rectangle over both the ventromedial nucleus of the PVN (PVN*vm*) and NTS and the square over the RVLM are ROIs in which measurements were made. 3v, third ventricle, cc, central canal, A, ambiguous nucleus. Scale bar = 50 μm. Graphs on the right depict FITC-10 kDa leakage values into the brain parenchyma of SHRs and Wistar groups within the PVN*vm*, NTS, and RVLM. Values are the means of 6–8 slices/rat, three rats/age/group. Comparisons made by two-way factorial ANOVA. PVN*vm*: *group* F (1,20) = 615 *P* < 0.001, *age* F (4,20) = 252 *P* < 0.001, *interaction* F (4,20) = 195 *P* < 0.001; NTS: *group* F (1,20) = 6305 *P* < 0.001, *age* F (4,20) = 1995 *P* < 0.001, *interaction* F (4,20) = 1750 *P* < 0.001; RVLM: *group* F (1,20) = 1293 *P* < 0.001, *age* F (4,20) = 323 *P* < 0.001, and *interaction* F (4,20) = 298 *P* < 0.001. Significances (*P* < 0.05): * vs. age-matched Wistar; † vs. respective week 4.

Sequential photomicrographs taken during the experimental protocol showed no detectable BBB leakage within the ventromedial PVN (PVN*vm*, an important autonomic integrative nucleus) of Wistar rats (Figure 2), a pattern also observed within the NTS and RVLM (data not shown). SHRs aged 4–5 weeks also exhibited no BBB leakage within the three autonomic areas, but a significant FITC extravasation to the brain parenchyma, initially observed at 6 weeks, increased continuously to 12 weeks of age. Quantitative data confirmed significant BBB leakage within the PVN*vm*, NTS, and RVLM in SHRs aged 6 weeks (Figure 2) preceding the significant pressure elevation by 2 weeks (Figure 1). Within the three autonomic nuclei, the BBB leakage (and MAP) increased progressively to the end of protocols. At the chronic phase of hypertension, FITC extravasation was higher in the PVN*vm*, with intermediate values in the NTS and minor leakage within the RVLM (graphs at right in Figure 2). From 4 weeks to 12 weeks of age, there were no significant changes in FITC leakage in age-matched normotensive controls.

**FIGURE 3:**
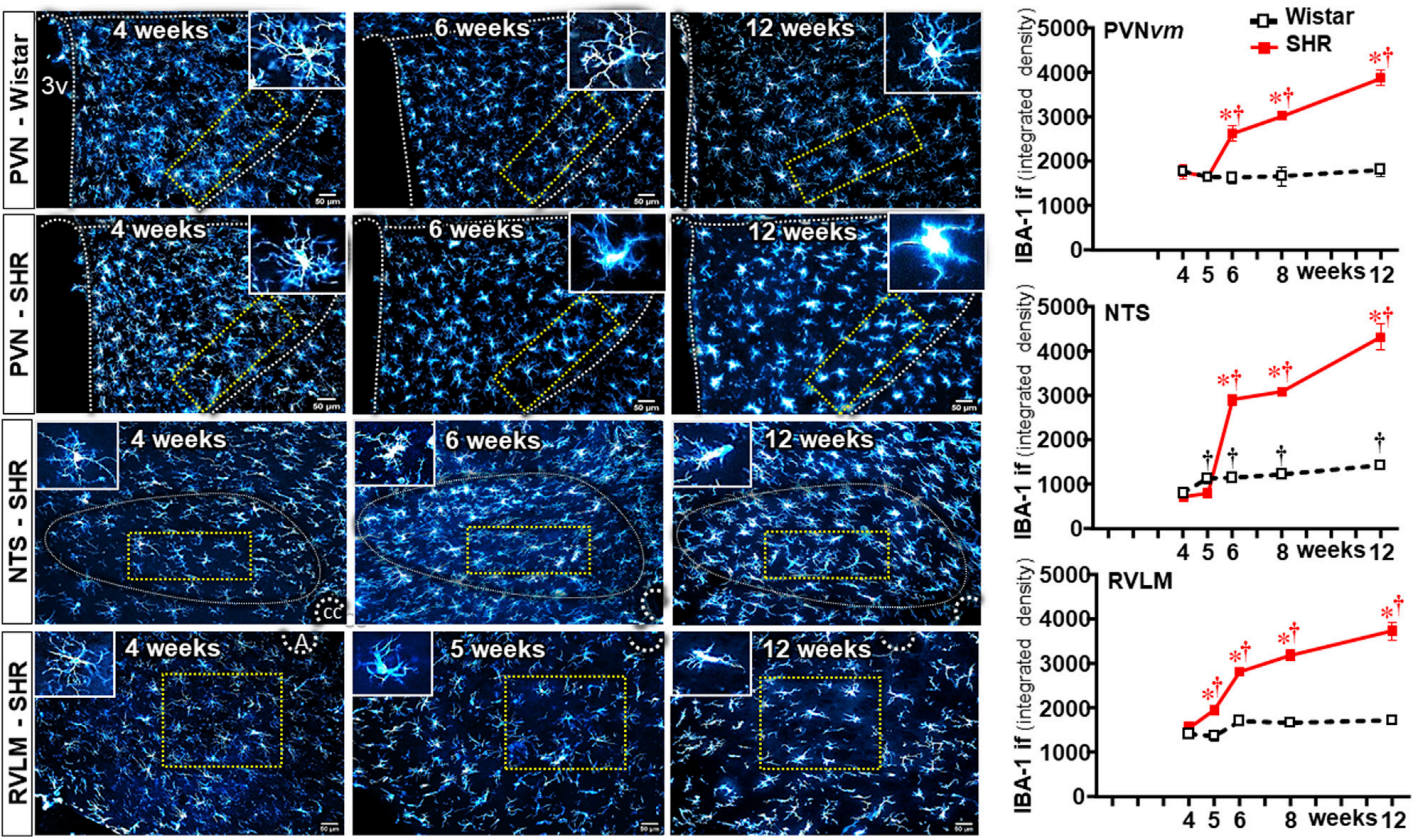
Microglia density changes within autonomic nuclei during the development of hypertension. Representative images of SHRs (PVN, NTS, and RVLM) and Wistar rats (PVN) aged 4 weeks, 6 weeks, and 12 weeks illustrate the morphological changes on microglial cells. The superimposed rectangle over both the ventromedial nucleus of the PVN (PVN*vm*) and the NTS and the square over the RVLM are ROIs in which measurements were made. Insets in the upper right or left corners show the morphology of a respective microglial cell in higher magnification. 3v, third ventricle, cc, central canal, A, ambiguous nucleus. Scale bar = 50 μm. Graphs on the right depict the values of microglia density of SHR and Wistar groups within the PVN*vm*, NTS, and RVLM. Values are the means of 6–8 slices/rat, 4–5 rats/age/group. Comparisons made by two-way factorial ANOVA. PVN*vm*: *group* F (1,40) = 101 *P* < 0.001, *age* F (4,40) = 23 *P* < 0.001, *interaction* F (4,40) = 21 *P* < 0.001; NTS: *group* F (1,40) = 1158 *P* < 0.001, *age* F (4,40) = 489 *P* < 0.001, *interaction* F (4,40) = 299 *P* < 0.001; RVLM: *group* F (1,34) = 469 *P* < 0.001, *age* F (4,34) = 141 *P* < 0.001, *interaction* F (4,34) = 138 *P* < 0.001. Significances (*P* < 0.05): * vs. age-matched Wistar; † vs. respective week 4.

We also analyzed the effects of establishing hypertension on microglia density and their morphological states as an index of changes from homeostatic-surveilling to disease-associated conditions in these groups of rats. Photomicrographs in Figure 3 depicted the changes in IBA-1 immunofluorescence in Wistar (PVN) rats and SHRs (PVN, NTS, and RVLM) during the experimental protocol. Quantitative data showed that microglia density/morphology within the PVN*vm* of Wistar rats was not altered during the experimental period, a pattern also observed in the RVLM (upper and lower graphs in Figure 3). Within the NTS, a mild increase in IBA-1 immunofluorescence was observed in Wistar rats from 5 weeks on (central graph in Figure 3). In contrast, SHRs aged 6–12 weeks exhibited a huge and progressive increase in IBA-1 immunofluorescence into the PVN*vm* and NTS; the RVLM also showed a significant increase in microglia density that occurred earlier in the 5th week. During the period of increasing BBB leakage and MAP elevation, IBA-1 density within the three autonomic nuclei augmented progressively, attaining very high values at the 12th week of age, indicating a higher protein availability within the microglial cells (Figure 3).

**FIGURE 4:**
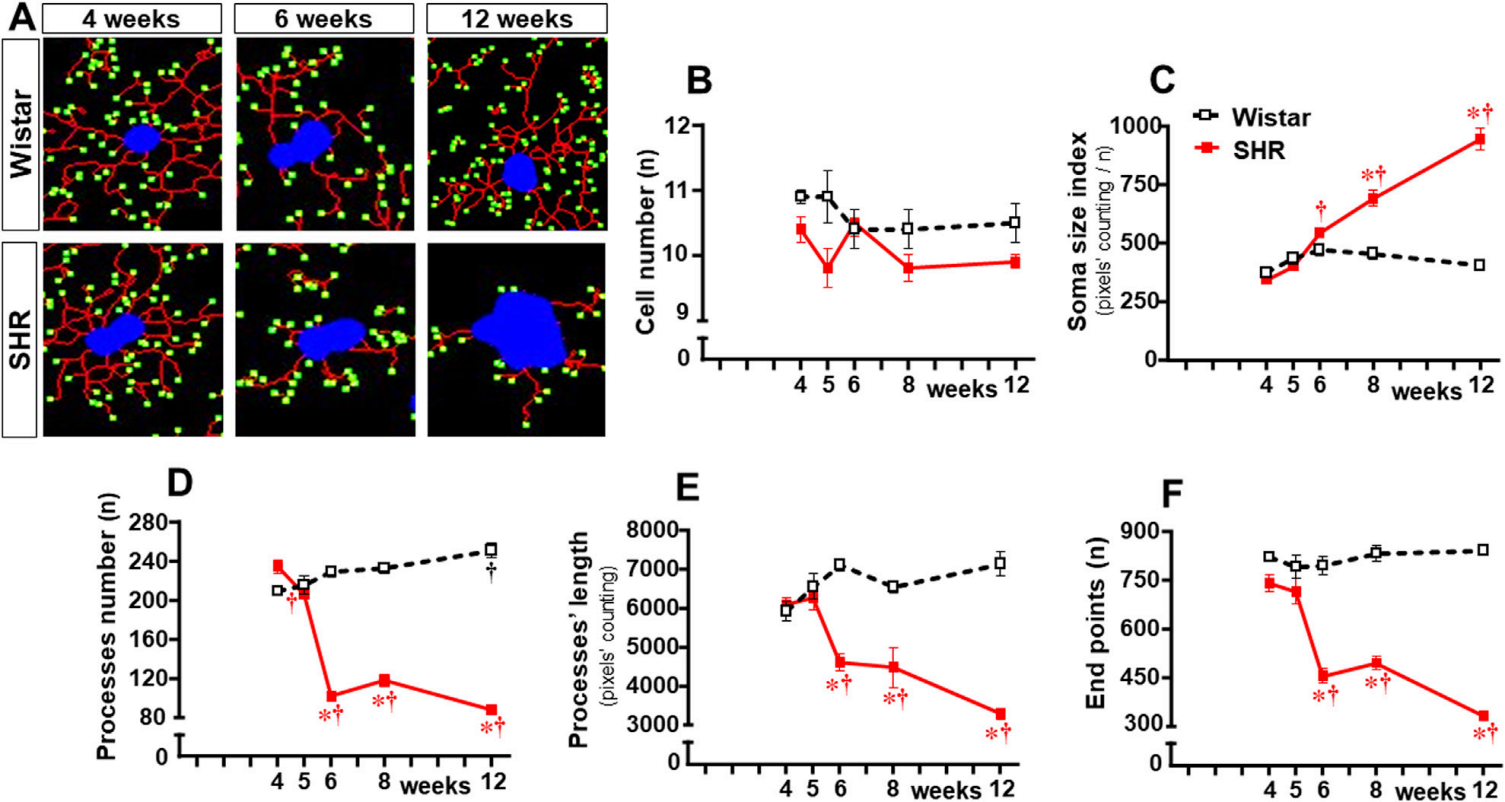
Morphological changes of the microglia within the PVN*vm* during the development of hypertension. **(A)**. Images of single microglial cells of the SHRs and Wistar rats aged 4 weeks, 6 weeks, and 12 weeks showing the soma size (blue), length and number of processes (red), and endpoints (green) that were obtained and quantified by the NeurphologyJ plugin to ImageJ. Graphs compare the sequential changes in cell number **(B)**, soma size index **(C)**, number of processes **(D)**, length of processes **(E)**, and endpoints **(F)** measured within the ROI superimposed over the PVN*vm*. n = 5 rats/age/group. Comparisons made by two-way factorial ANOVA. Cell number: *group* F (1,40) = 11,1 *P* = 0.002, *age* F (4,40) = 1.4 *P* = 0.251, *interaction* F (4,40) = 1.4 *P* = 0.256; Soma size index: *group* F (1,40) = 92.0 *P* < 0.001, *age* F (4,40) = 46.6 *P* < 0.001, *interaction* F (4,40) = 42.9 *P* < 0.001; number of processes: *group* F (1,40) = 475.0 *P* < 0.001, *age* F (4,40) = 41.7 *P* < 0.001, *interaction* F (4,40) = 102.6 *P* < 0.001; length of processes: *group* F (1,40) = 92.2 *P* < 0.001, *age* F (4,40) = 5.32 *P* = 0.002, *interaction* F (4,40) = 17.1 *P* < 0.001; endpoints: *group* F (1,40) = 271.6 *P* < 0.001, *age* F (4,40) = 20.8 *P* < 0.001, *interaction* F (4,40) = 26.4 *P* < 0.001. Significances (*P* < 0.05): * vs. age-matched Wistar; † vs. respective week 4.

**FIGURE 5:**
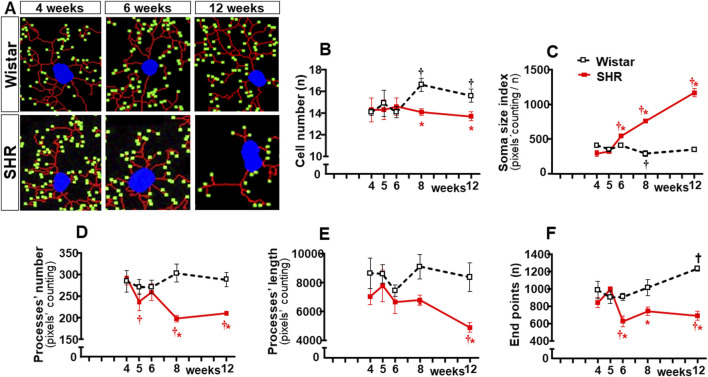
Morphological changes of microglia within the NTS during the development of hypertension. **(A)**. Images of single microglial cells of SHRs and Wistar rats aged 4 weeks, 6 weeks, and 12 weeks showing the soma size (blue), number and length of processes (red), and endpoints (green) were obtained and quantified by the NeurphologyJ plugin to ImageJ. Graphs compare the sequential changes in cell number **(B)**, soma size index **(C)**, number of processes **(D)**, length of processes **(E)**, and endpoints **(F)** within the NTS of the SHR and Wistar groups during the development of hypertension. n = 5 rats/age/group. Comparisons made by two-way factorial ANOVA. Cell number: *group* F (1,40) = 17.09 *P* < 0.001, *age* F (4,40) = 3.62 *P* = 0.013, *interaction* F (4,40) = 7.87 *P* < 0.001; Soma size index: *group* F (1,40) = 679.2 *p* < 0.001, *age* F (4,40) = 237.0 *p* < 0.001, *interaction* F (4,40) = 303.7 *P* < 0.001; number of processes: *group* F (1,40) = 81.67 *P* < 0.001, *age* F (4,40) = 8.76 *P* < 0.001, *interaction* F (4,40) = 18.20 *P* < 0.001; length of processes: *group* F (1,40) = 70.89 *P* < 0.001, *age* F (4,40) = 7.57 *P* < 0.001, *interaction* F (4,40) = 5.51 *P* = 0.001; Endpoints: *group* F (1,40) = 161.9 *P* < 0.001, *age* F (4,40) = 14.93 *P* < 0.001, *interaction* F (4,40) = 32.47 *P* < 0.001. Significances (*P* < 0.05): * vs. age-matched Wistar; † vs. respective week 4.

**FIGURE 6:**
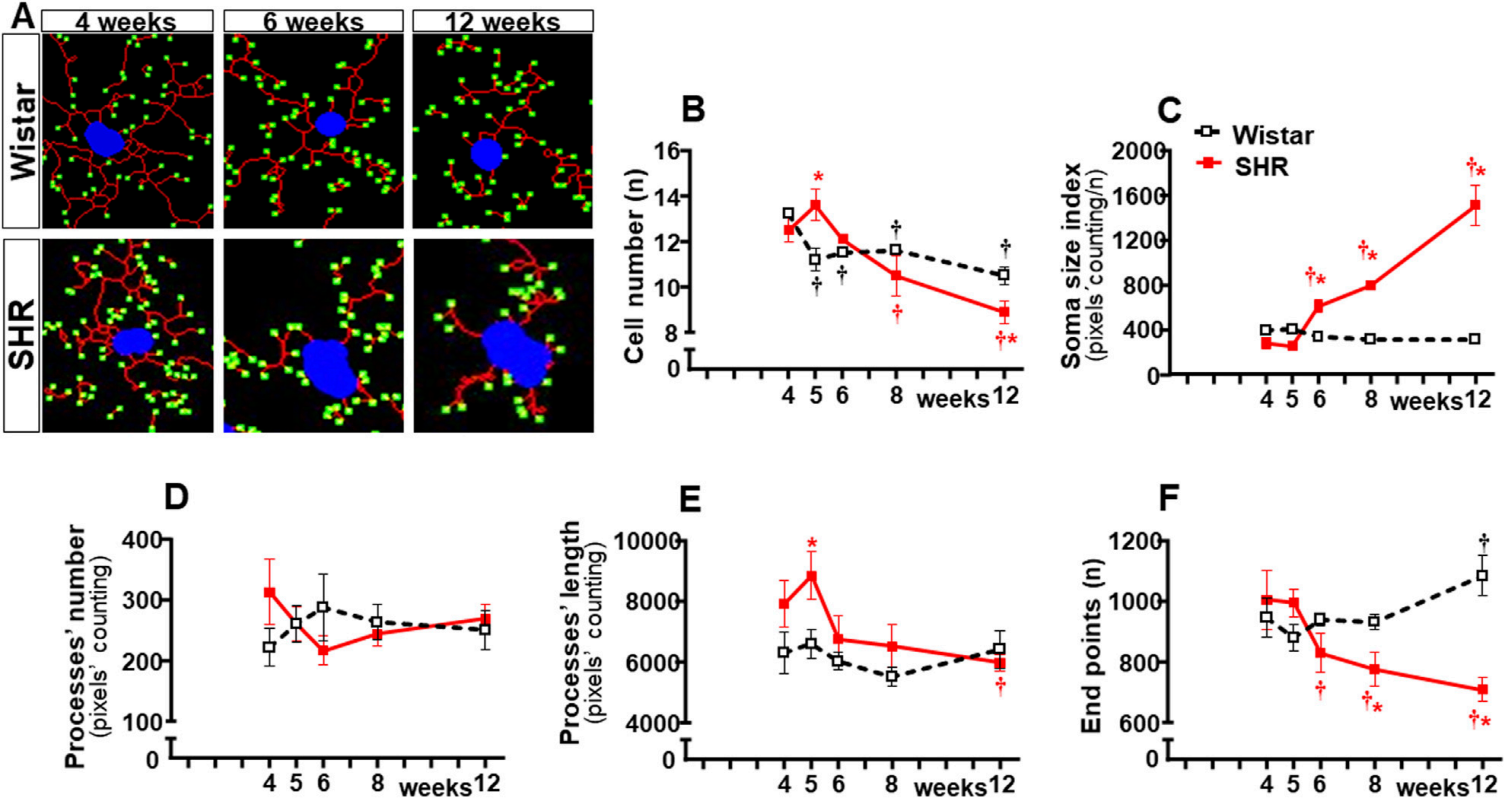
Morphological changes of microglia within the RVLM during the development of hypertension. **(A)**. Images of single microglial cells of SHRs and Wistar rats aged 4 weeks, 6 weeks, and 12 weeks showing the soma size (blue), number and length of processes (red), and endpoints (green) were obtained and quantified by the NeurphologyJ plugin to ImageJ. Graphs compare the sequential changes in cell number **(B)**, soma size index **(C)**, number of processes **(D)**, length of processes **(E)**, and endpoints **(F)** within the RVLM of SHRs and Wistar groups during the development of hypertension. n = 4-5 rats/age/group. Comparisons made by two-way factorial ANOVA. Cell number: *group* F (1,34) = 0.34 *P* = 0.562, *age* F (4,34) = 65.67 *P* < 0.001, *interaction* F (4,34) = 27.64 *P* < 0.001; Soma size index: *group* F (1,34) = 300.6 *P* < 0.001, *age* F (4,34) = 119.9 *P* < 0.001, *interaction* F (4,34) = 158.7 *P* < 0.001; number of processes: *group* F (1,34) = 0.17 *P* = 0.683, *age* F (4,34) = 0.30 *P* = 0.878, *interaction* F (4,34) = 7.37 *P* = 0.005; length of processes: *group* F (1,34) = 35.96 *P* < 0.001, *age* F (4,34) = 13.73 *P* < 0.001, *interaction* F (4,34) = 6.78 *P* < 0.001; Endpoints: *group* F (1,34) = 35.32 *P* < 0.001, *age* F (4,34) = 7.21 *P* < 0.001, *interaction* F (4,34) = 29.85 *P* < 0.001. Significances (*P* < 0.05): * vs. age-matched Wistar; † vs. respective week 4.

In contrast to Wistar rats, in which microglial cell morphology did not show visible changes throughout the experimental period, drastic morphologic changes were observed in SHRs during the establishment of hypertension (compare the amplified images on the insets of Figure 3). Therefore, we used NeurphologyJ (an automatic freeware tool) to quantify the 2D microglia fluorescent images and analyze the effects of hypertension. Figure 4A depicts the morphology of single PVN microglial cells representative of their groups at different ages, showing the soma size, the processes branching emerging from the soma, and the numerous endpoints of these microglial processes. The number of microglial cells within the PVN*vm* was similar in the SHR and Wistar groups aged 4–12 weeks (Figure 4B), but the soma size of the SHRs exhibited a progressive increase starting at the 6th week (Figure 4C). SHRs also showed a slight decrease in the number of microglial processes in the 5th week, with a large reduction from 6 weeks to 12 weeks, which was accompanied by a marked reduction in the length of the processes and a great decrease in microglial endpoints (Figures 4D–F, respectively). During the experimental period, the number of microglial cells did not change within the NTS of the SHRs, while a small but significant increase was observed in Wistar rats aged 8 and 12 weeks (Figures 5A, B). Similar to that observed in the PVN*vm*, the decrease in the number of processes of NTS microglial cells started at the 5th week, while the soma size increased progressively from 6 weeks to 12 weeks (Figure 5D, C respectively). The length of the microglia processes (12 weeks) and the endpoints within the NTS (6–12 weeks) also decreased in the SHR group (Figures 5E, F). In accordance with the PVN*vm* and NTS, the RVLM microglial cells in SHRs aged 6 weeks to 12 weeks also exhibited a progressive increase in soma size accompanied by a sequential decrease in endpoints (Figures 6A, C, F). However, opposite to the other two autonomic nuclei, the numbers of microglia cells within the RVLM diminished in both groups, while the number of microglia processes of the SHRs did not change during the experimental protocol (Figures 6B, D). SHRs also exhibited a late decrease in RVLM microglial process length (12 weeks, Figure 6E).

### Sequential changes of Ang II density within autonomic areas

**FIGURE 7:**
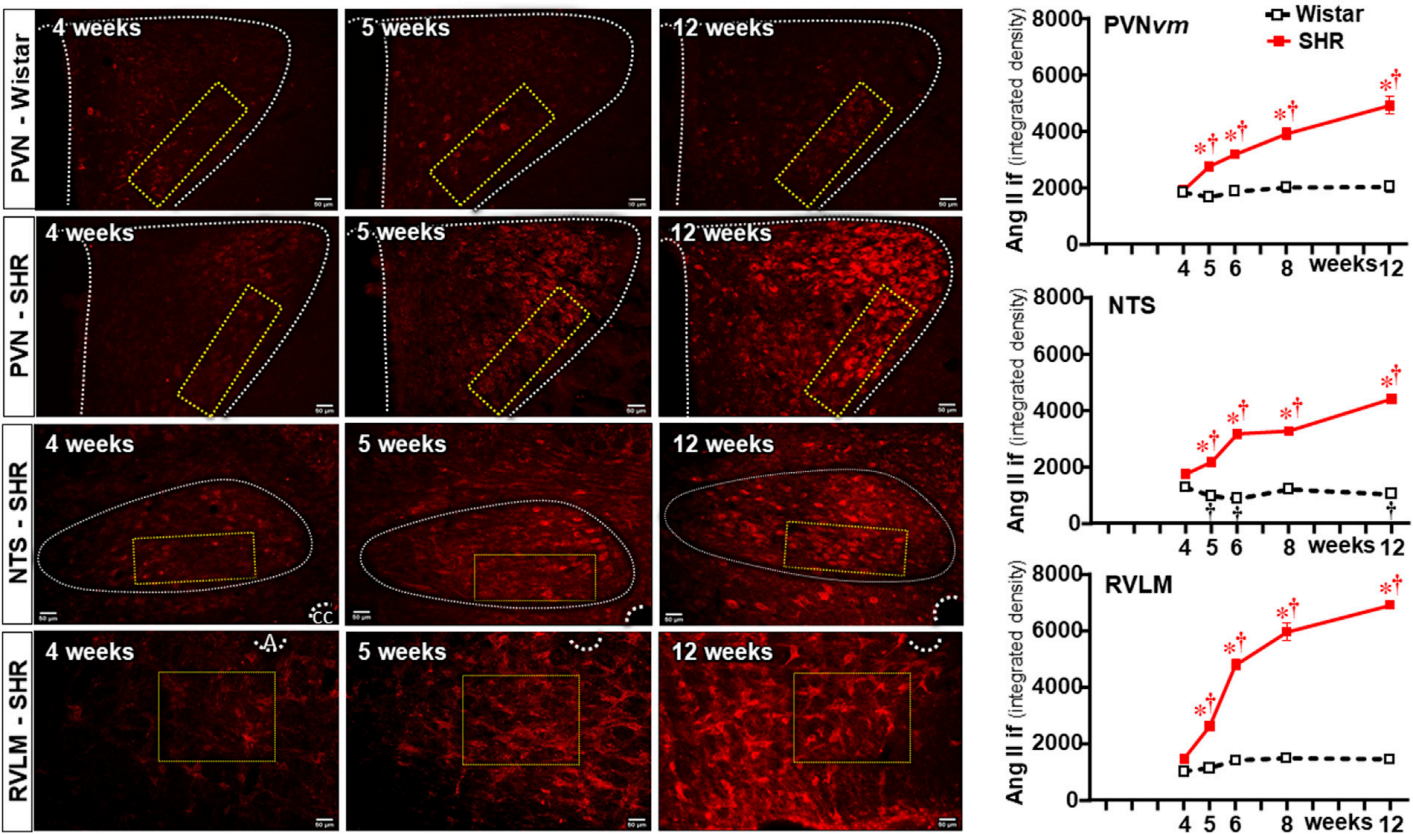
Changes of Ang II density within autonomic nuclei during the development of hypertension. Representative images of SHRs (PVN, NTS, and RVLM) and Wistar rats (PVN) aged 4 weeks, 5 weeks, and 12 weeks depict Ang II availability in these nuclei. The superimposed rectangle over both the ventromedial nucleus of the PVN (PVN*vm*) and NTS and the square over the RVLM are the ROIs in which measurements were made. 3v, third ventricle, cc, central canal, A, ambiguous nucleus. Scale bar = 50 μm. Graphs on the right depict the values of Ang II density within the PVN*vm*, NTS, and RVLM. Values are the means of 6–8 slices/rat, 4–5 rats/age/group. Comparisons made by two-way factorial ANOVA. PVN*vm*: *group* F (1,40) = 184.6 *P* < 0.001, *age* F (4,40) = 27.8 *P* < 0.001, *interaction* F (4,40) = 18.4 *P* < 0.001; NTS: *group* F (1,40) = 4403 *P* < 0.001, *age* F (4,40) = 248.4 *P* < 0.001, *interaction* F (4,40) = 307.2 *P* < 0.001; RVLM: *group* F (1,34) = 1040.0 *P* < 0.001, *age* F (4,34) = 133.0 *P* < 0.001, *interaction* F (4,34) = 93.6 *P* < 0.001. Significances (*P* < 0.05): * vs. age-matched Wistar; † vs. respective week 4.

Because previous studies from our and other groups have linked Ang II availability with both BBB dysfunction and microglia activation (Biancardi et al., 2014; Buttler et al., 2017; Mowry et al., 2021), we also analyzed the time-course changes of Ang II density within the three autonomic nuclei during the establishment of hypertension in the same ROIs of SHRs and Wistar rats. Sequential photomicrographs taken during the experimental protocol showed that Ang II immunofluorescence did not change within the PVN*vm* of Wistar rats (Figure 7). Similar immunofluorescence was also observed in SHRs aged 4 weeks, but SHRs exhibited progressive increases from the 5th week on. Quantitative data confirmed similar Ang II density within the three autonomic nuclei of SHRs and Wistar rats aged 4 weeks (graphs at right in Figure 7). In contrast, SHRs showed a precocious increase in Ang II density at the 5th week, with progressive augmentation at 6 weeks, 8 weeks, and 12 weeks (Figure 7). Except for small decreases within the NTS, Ang II immunoreactivity did not change in the Wistar group during the experimental period. Notice that during the establishment of hypertension, Ang II immunofluorescence attained a higher value in the RVLM (6931 ± 84 A.U.) than in the PVN*vm* and NTS (4938 ± 301 A. U. and 4417 ± 124 A.U., respectively).

**FIGURE 8:**
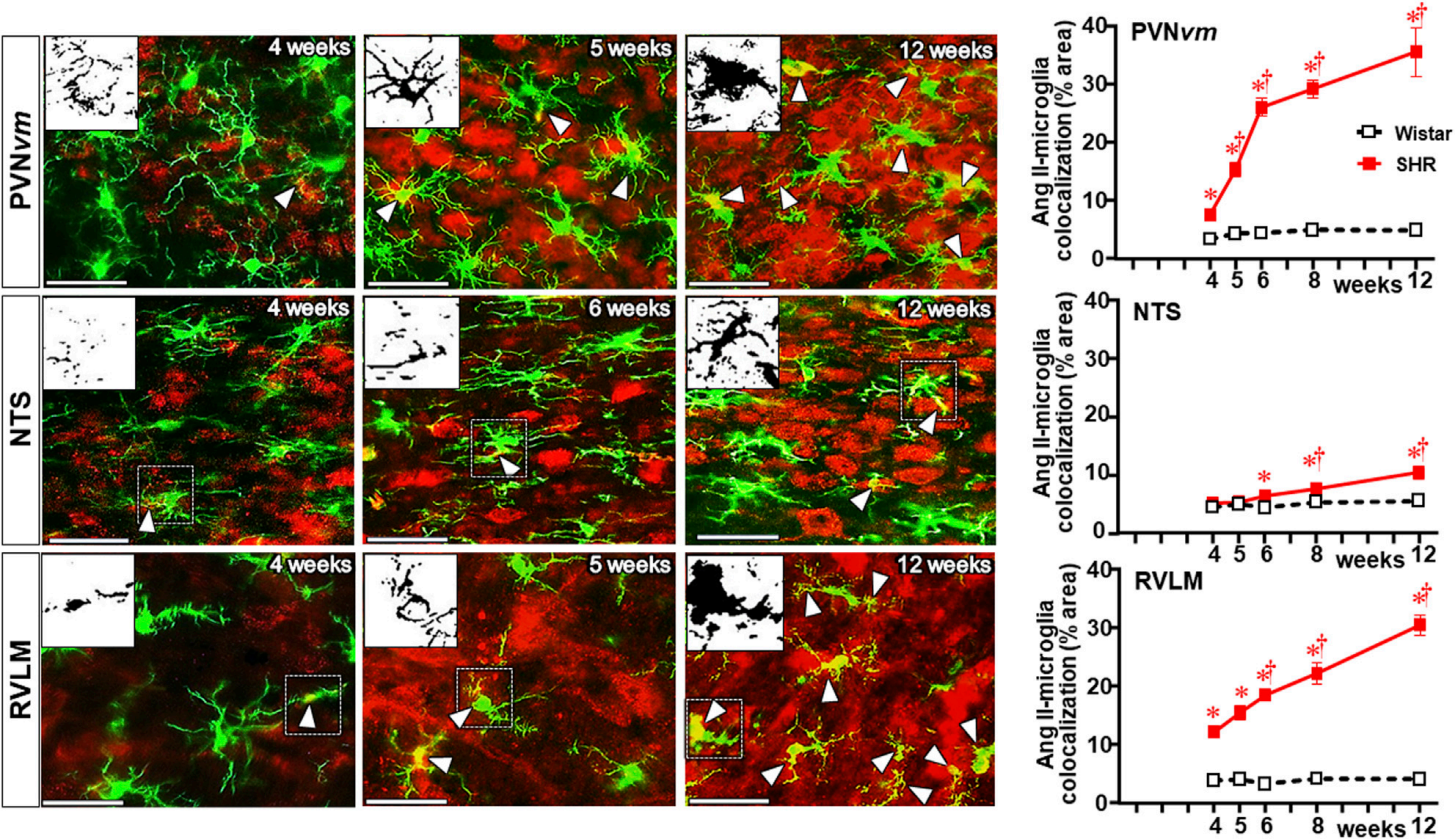
Colocalization of Ang II with microglia within the autonomic nuclei during the development of hypertension. Images show Ang II immunofluorescence (red), microglia (green), and their colocalization (yellow) in both soma and processes (white arrowheads) within the PVN*vm*, NTS, and RVLM of SHRs aged 4 weeks, 5 weeks, and 12 weeks. Magnified binary images in insets in the upper left corners depict Ang II-IBA-1 colocalization within the soma and processes of a microglial cell (small, dashed square) and also show the intensification of Ang II-IBA-1 colocalization during the experimental protocol. Scale bar = 50 μm. Graphs at the right depict the quantitative analysis of Ang II-microglia colocalization into specified ROIs of PVN*vm*, NTS, and RVLM during the experimental protocol in the SHR and Wistar groups. n = 4–5 rats/age/group. Comparisons made by two-way factorial ANOVA. PVN*vm*: *group* F (1,40) = 342.6 *P* < 0.001, *age* F (4,40) = 28.1 *P* < 0.001, *interaction* F (4,40) = 23.1 *P* < 0.001; NTS: *group* F (1,40) = 25.4 *P* < 0.001, *age* F (4,40) = 7.9 *P* < 0.001, *interaction* F (4,40) = 3.7 *P* = 0.013; RVLM: *group* F (1,34) = 575.1 *P* < 0.001, *age* F (4,34) = 24.5 *P* < 0.001, *interaction* F (4,34) = 22.5 *p* < 0.001. Significances (*P* < 0.05): * vs. age-matched Wistar; † vs. respective week 4.

Double immunostaining for Ang II and IBA-1 allowed us to analyze the colocalization of both. Photomicrographs in Figure 8 depict Ang II-microglia association within the three autonomic areas of the SHRs, showing mild colocalization with few microglial cells at the pre-hypertensive phase, with a considerable increase after the 5th–6th week of age, when it was strong and present in a large number of cells. Insets in the photomicrographs depict the binary colocalized images showing not only Ang II association with soma and processes of microglial cells but also the IBA-1 protein availability that augmented from the beginning to the end of the experimental protocol. Quantitative data confirmed minimal colocalization within the three autonomic nuclei of Wistar rats (average of 3% area) with no change during the experimental protocol (graphs at right in Figure 8). Compared to normotensive controls, there was a significant increase in Ang II-microglia colocalization into the PVN and RVLM after the 4th–5th week (8%–14% area), which augmented progressively after BBB disruption, attaining very high values (average of 30%–35% area) in SHRs aged 12 weeks (upper and lower graphs in Figure 8). Interestingly, despite the high Ang II availability in the NTS of SHRs (Figure 7), its colocalization with microglial cells was very low and similar to normotensive controls between the 4th and the 5th weeks, showing only small increases at the 8th and 12th weeks (8% and 10% area, respectively (central graph in Figure 8).

### Sequential changes on BBB function, microglia, and Ang II densities within non-autonomic areas

**FIGURE 9:**
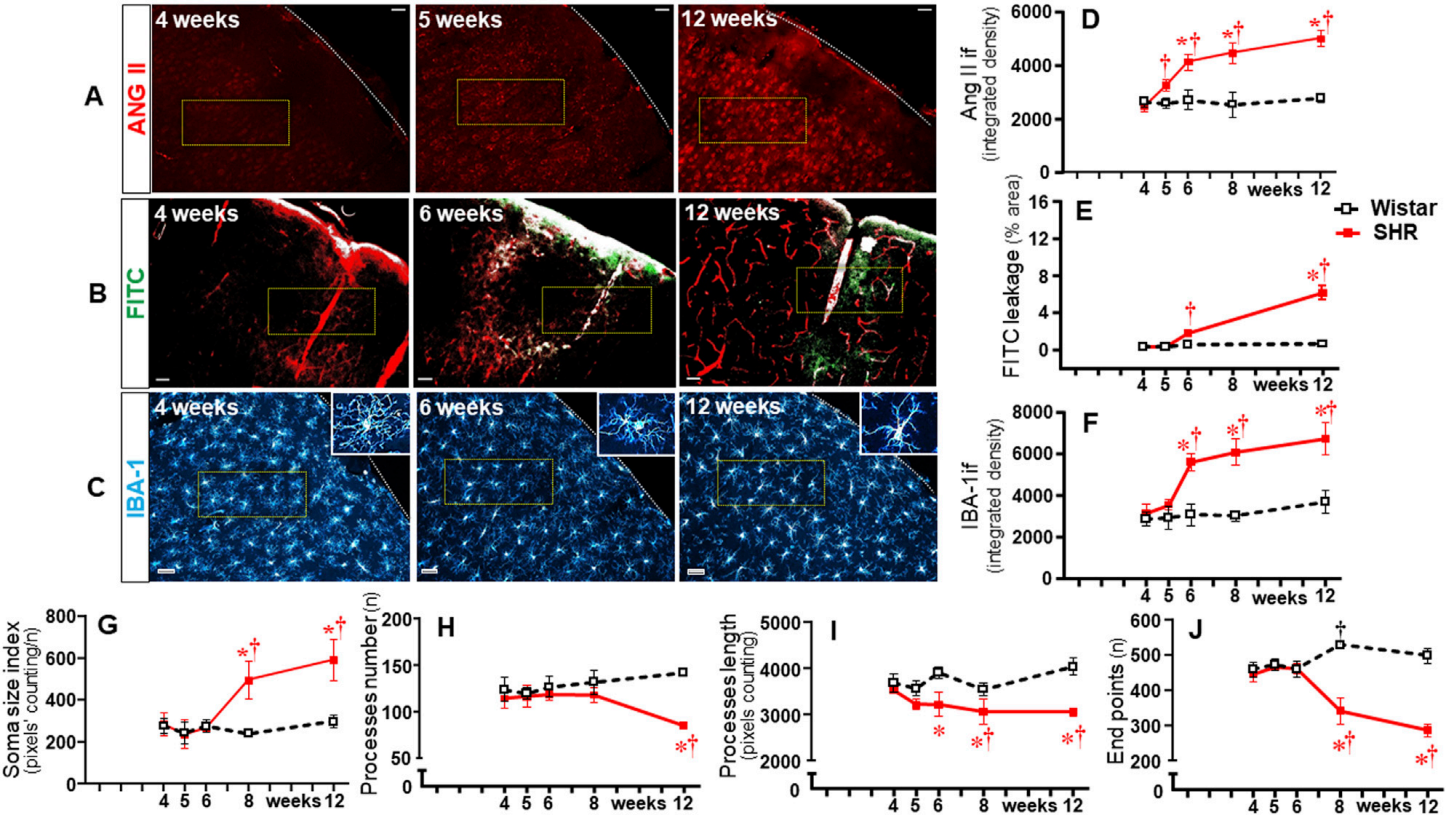
Time-course changes of Ang II immunofluorescence, BBB permeability, microglia density, and their morphological states within the somatosensory cortex during the development of hypertension. Images of SHRs aged 4 weeks, 5 weeks, 6 weeks, and 12 weeks show Ang II availability **(A)**, FITC leakage **(B)**, and microglia immunofluorescence (**C**-insets in the right upper corner show the morphology of a respective microglial cell in higher magnification); Scale bar = 50 μm. The superimposed rectangle indicates the ROI in which measurements were made. Graphs compare the temporal changes on Ang II immunofluorescence **(D)**, BBB permeability **(E)**, microglia density **(F)**, soma size **(G)**, number of processes **(H)**, length of processes **(I)**, and endpoints **(J)** of microglial cells of the SHR and Wistar groups during the experimental protocol. Values are the means of 6–8 slices/rat, 3–4 rats/age/group. Comparisons made by two-way factorial ANOVA. Ang II density: *group* F (1,30) = 140.1 *P* < 0.001, *age* F (4,30) = 21.81 *P* < 0.001, *interaction* F (4,30) = 18.74 *P* < 0.001; FITC leakage: *group* F (1,16) = 195.5 *P* < 0.001, *age* F (3,16) = 142.8 *P* < 0.001, *interaction* F (3,16) = 120.3 *P* < 0.001; microglia density: *group* F (1,30) = 118.6 *P* < 0.001, *age* F (4,30) = 23.01 *P* < 0.001, *interaction* F (4,30) = 11.98 *P* < 0.001; soma size index: *group* F (1,30) = 35.97 *P* < 0.001, *age* F (4,30) = 17.54 *P* < 0.001, *interaction* F (4,30) = 14.32 *P* < 0.001; number of processes: *group* F (1,30) = 36.31 *P* < 0.001, *age* F (4,30) = 1.55 *P* = 0.213, *interaction* F (4,30) = 9.90 *P* < 0.001; length of processes: *group* F (1,30) = 85.78 *P* < 0.001, *age* F (4,30) = 4.04 *P* = 0.010, *interaction* F (4,30) = 6.61 *P* = 0.001; Endpoints: *group* F (1,30) = 138.6 *P* < 0.001, *age* F (4,30) = 14.59 *P* < 0.001, *interaction* F (4,30) = 45.90 *P* < 0.001. Significances (*P* < 0.05): * vs. age-matched Wistar; † vs. respective week 4.

**FIGURE 10:**
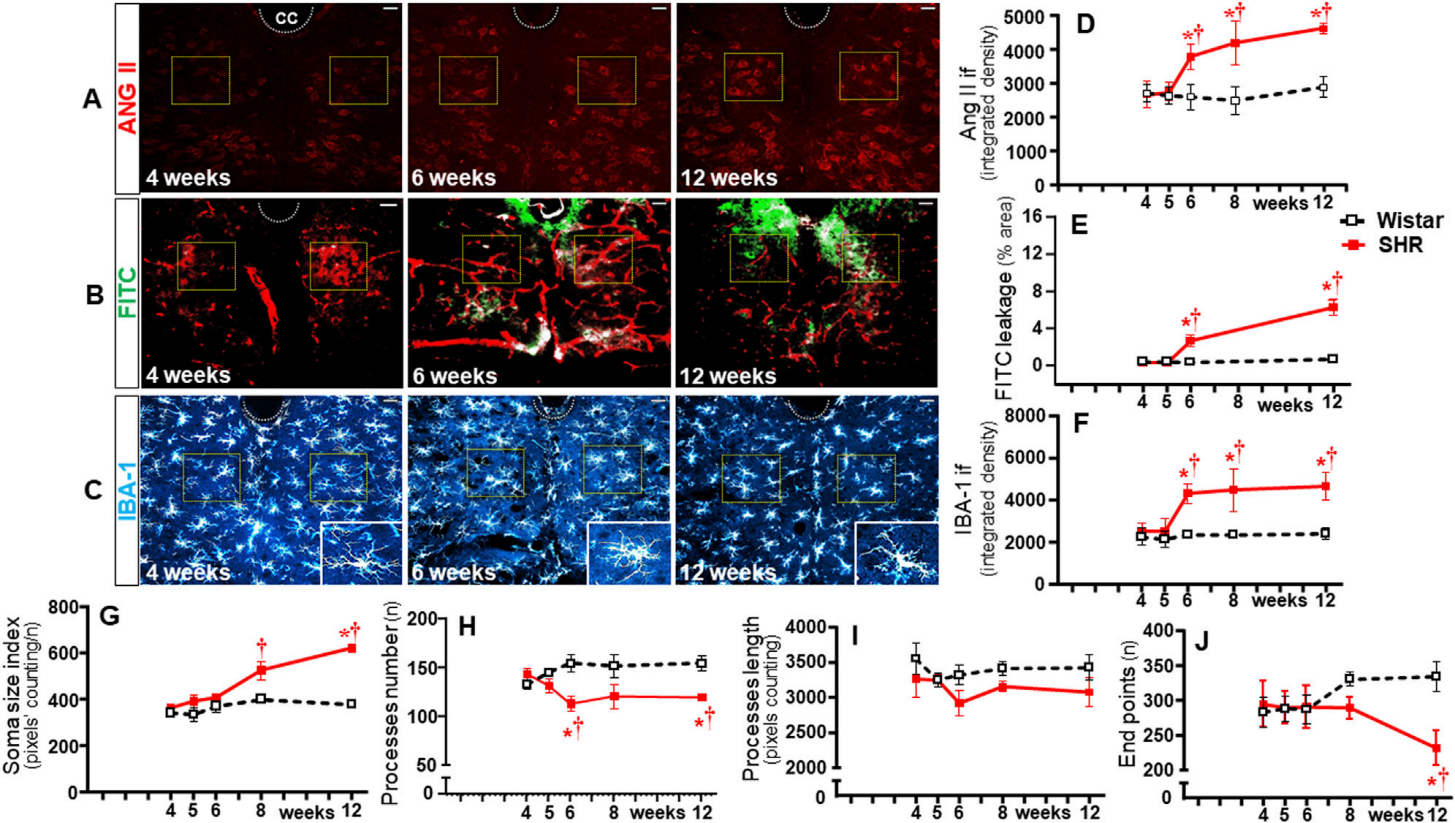
Time-course changes of Ang II immunofluorescence, BBB permeability, microglia density, and their morphological states within the hypoglossal nucleus during the development of hypertension. Images of SHRs aged 4 weeks, 6 weeks, and 12 weeks show Ang II availability **(A)**, FITC leakage **(B)**, and microglia immunofluorescence **(C)**-insets in the lower right corner show the morphology of a respective microglial cell in higher magnification). Scale bar = 50 μm. The superimposed squares indicate the ROIs in which measurements were made. Graphs compare the temporal changes on Ang II immunofluorescence **(D)**, BBB permeability **(E)**, microglia density **(F)**, soma size **(G)**, number of processes **(H)**, length of processes **(I)**, and endpoints **(J)** of microglial cells of the SHR and Wistar groups during the experimental protocol. Values are the means of 6–8 slices/rat, 3–4 rats/age/group. Comparisons made by two-way factorial ANOVA. Ang II density: *group* F (1,30) = 61.82 *P* < 0.001, *age* F (4,30) = 12.80 *P* < 0.001, *interaction* F (4,30) = 10.42 *P* < 0.001; FITC leakage: *group* F (1,16) = 142.1 *P* < 0.001, *age* F (3,16) = 83.36 *P* < 0.001, *interaction* F (3,16) = 69.49 *P* < 0.001; microglia density: *group* F (1,30) = 65.74 *P* < 0.001, *age* F (4,30) = 10.10 *P* < 0.001, *interaction* F (4,30) = 7.00 *P* = 0.001; soma size index: *group* F (1,30) = 34.57 *P* < 0.001, *age* F (4,30) = 12.39 *P* < 0.001, *interaction* F (4,30) = 5.98 *P* = 0.001; number of processes: *group* F (1,30) = 20.55 *P* < 0.001, *age* F (4,30) = 0.11 *P* = 0.979, *interaction* F (4,30) = 3.46 *P* = 0.021; length of processes: *group* F (1,30) = 24.90 *P* < 0.001, *age* F (4,30) = 2.91 *P* = 0.004, *interaction* F (4,30) = 2.13 *P* = 0.104; endpoints: *group* F (1,30) = 10.74 *P* = 0.003, *age* F (4,30) = 1.28 *P* = 0.303, *interaction* F (4,30) = 7.65 *P* = 0.001. Significances (*P* < 0.05): * vs. age-matched Wistar; † vs. respective week 4.

**FIGURE 11:**
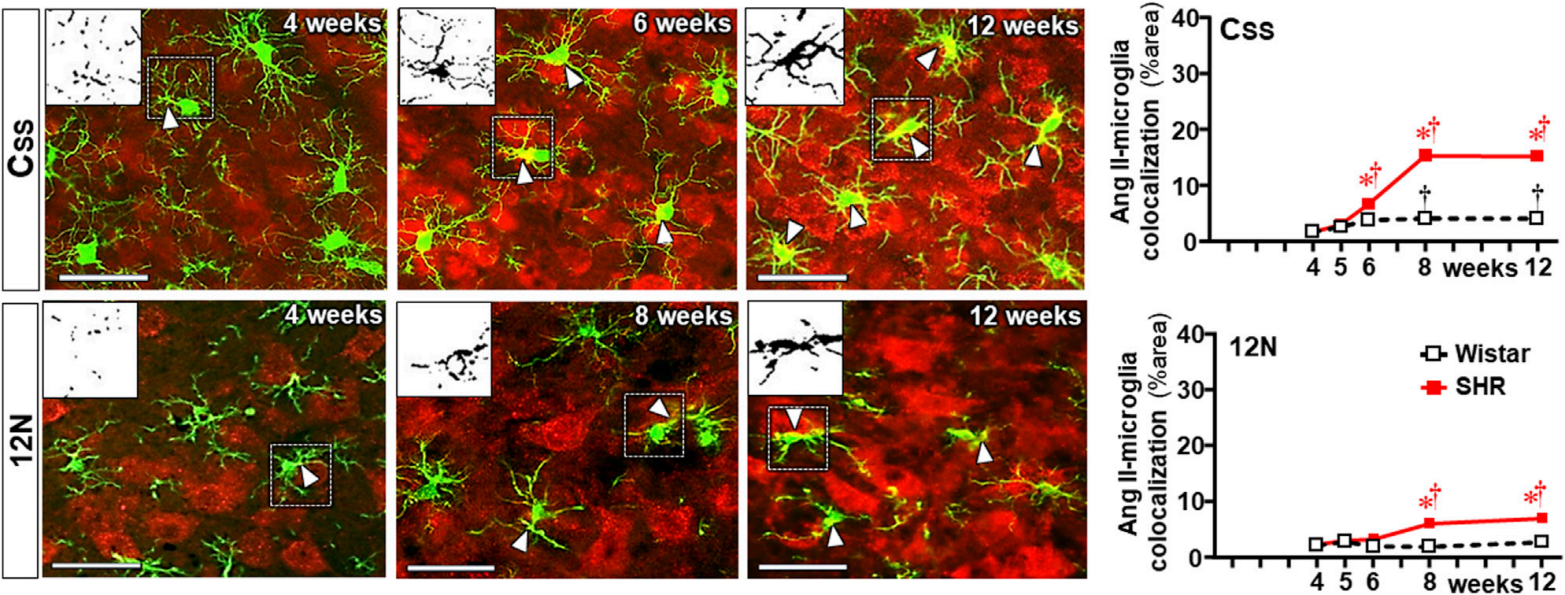
Colocalization of angiotensin II and microglia within the somatosensory cortex (CSS) and hypoglossal nucleus (12N) during the development of hypertension. Images show angiotensin II immunofluorescence (red), microglia (green), and their colocalization (yellow) in both soma and processes (white arrowheads) in SHRs aged 4 weeks, 6 weeks or 8 weeks, and 12 weeks. Magnified binary images in the upper left corner insets depict Ang II-IBA-1 colocalization within soma and processes of a microglial cell (small, dashed square) showing, in addition, the intensification of Ang II-IBA-1 colocalization during the experimental protocol. Scale bar = 50 μm. Values are the means of 6–8 slices/rat, 3–4 rats/age/group. Comparisons made by two-way factorial ANOVA. C_SS_: *group* F (1,30) = 712.4 *P* < 0.001, *age* F (4,30) = 313.4 *P* < 0.001, *interaction* F (4,30) = 173.3 *P* < 0.001; 12N: *group* F (1,30) = 80.7 *P* < 0.001, *age* F (4,30) = 20.3 *P* < 0.001, *interaction* F (4,30) = 17.0 *P* < 0.001. Significances (*P* < 0.05): * vs. age-matched Wistar; † vs. respective week 4.

To identify whether observed changes were specific to autonomic areas, we also analyzed the effects of establishing hypertension within the C_SS_ (Figure 9) and 12N (Figure 10) in the same rats. In both nuclei of normotensive rats, Ang II density did not change during the experimental protocol, while a significant augmentation was observed in SHRs at the 5th week (C_SS_, Figures 9A, D) and at the 6th week (12N, Figures 10A, D), with further increases to the 12th week of age. Increased BBB permeability and microglia density within the C_SS_ and 12N were only observed in SHRs aged 6–12 weeks (panels **B**, **C**, **E**, and **F** in Figures 9, 10). The number of microglial cells in C_SS_ and 12N was similar in both strains and did not change during the experimental period. On the other hand, SHRs exhibited increased soma size and reduced endpoints during the establishment of hypertension (panels **C**, **G**, and **J** in Figures 9, 10). These changes, however, appeared later than those observed within the autonomic nuclei. In contrast to autonomic areas (in which the number and length of microglial processes showed an early and robust decrease during the transition from the pre- to the hypertensive phase), the elevation of pressure levels was accompanied by only a small reduction or no change in the number and length of microglial processes (panels C, H and I in Figures 9, 10). None of these changes were observed within the C_SS_ and 12N of normotensive rats. Non-autonomic areas of SHRs also exhibited a late and reduced Ang II-microglia colocalization, starting at the 6th (C_SS_ = 7% area) and 8th weeks (12N = 6% area) and increasing to the end of the protocol (15% and 7% area, respectively, Figure 11).

### Comparison of autonomic vs. non-autonomic effects

Although BBB leakage, microglia changes, increased Ang II density, and Ang II-microglia association occurred in both autonomic and non-autonomic areas during the establishment of hypertension, the magnitudes of responses were quite different between these areas. To better evaluate these differences, we calculate the changes observed from the pre- (4th week) to the hypertensive phase (12th week) for all measured variables. PVN*vm*, NTS, RVLM, CSS, and 12N data are presented in Table 1. There were slight differences between the autonomic nuclei, but except for IBA-1 density, the increases in Ang II availability, BBB permeability, Ang II-microglia colocalization, and soma size of microglial cells, as well as the reductions in microglia process number/length and endpoints observed in these nuclei, were always higher than those exhibited by non-autonomic areas (Table 1).

**TABLE 1:** Angiotensin II immunofluorescence (Ang II *if*), blood–brain barrier (BBB) leakage, microglial cell density (IBA-1 *if*), and Ang II-IBA-1 colocalization changes observed within autonomic and non-autonomic areas from the beginning to the end of the experimental protocol. The morphological changes of microglial cells during the establishment of hypertension are also shown.

	PVN*vm*	NTS	RVLM	CSS	12N
Ang II *if* (integrated density)	3002 ± 178	2670 ± 158	5460 ± 55*^#^	2222 ± 2*^#^	2698 ± 175*^#^
BBB leakage (% area)	15.39 ± 0.50	10.70 ± 0.19*	7.31 ± 0.25*†	5.78 ± 0.25*†	6.10 ± 0.35*†
IBA-1 *if* (integrated density)	2245 ± 88	3603 ± 282*	2183 ± 105†	2968 ± 350	2833 ± 403
Ang II-IBA-1 colocalization (% area)	27.2 ± 4.6	5.1 ± 1.4*^#^	20.4 ± 1.5	13.6 ± 0.5*	5.2 ± 0.8*^#^
Microglial cells					
Cell number (n)	no change	no change	no change	no change	no change
Soma size index (pixels counting/n)	604 ± 37	837 ± 70	1226 ± 202*	358 ± 81*†^#^	153 ± 55*†^#^
Number of processes(n)	−161 ± 15	−81 ± 18*	−71 ± 15*	−54 ± 20*	−20 ± 4*†
Length of processes (number of pixels)	−3476 ± 423	−2595 ± 588	−2742 ± 570	−638 ± 166*^#^	−641 ± 67*^#^
Endpoints (n)	−493 ± 61	−99 ± 44*^#^	−378 ± 94	−265 ± 14	−92 ± 6*

Values represent the magnitude of changes observed in SHRs from 4 weeks to 12 weeks of age. *if*, immunofluorescence. Comparisons made by one-way ANOVA. Significances (*P* < 0.05) are * vs. PVN, † vs. NTS, # vs. RVLM.

## Discussion

The present set of data corroborates previous findings on BBB dysfunction, microglia activation, and autonomic imbalance in hypertension. Additionally, by analyzing respective time-course changes during the transition from pre-to hypertensive phase, new original observations were made: 1) BBB leakage and microglia activation occur almost simultaneously within autonomic nuclei of the SHRs preceding both pressure elevation and autonomic imbalance; 2) an initial increase of local Ang II density precedes these responses; 3) from the 5th to 6th week on, BBB leakage, Ang II availability, and IBA-1 density augment continuously, allowing a parallel increase in both Ang II-microglia colocalization and the transition of microglial cells from highly ramified to short process arbors, fewer endpoints and enlarged soma; 4) simultaneously with increased Ang II-microglia colocalization and morphologic phenotypic changes of microglial cells, the vasomotor sympathetic activity augments, the autonomic control deteriorates, and the blood pressure increases; 5) both the increased Ang II availability (early brain change followed by leaked plasma accumulation) preceding BBB leakage and microglia activation in SHRs and the absence of these responses in normotensive rats in which there is no alteration in Ang II availability suggest a cause–effect relationship; 6) lower responses are also observed in sensory and motor integrative brain areas, suggesting they are not specific but indicate a clear predominance of hypertension-induced effects on autonomic nuclei.

BBB dysfunction and altered microglial states have been reported in neurodegenerative, traumatic, and inflammatory diseases. Studies have advanced considerably in this field, showing BBB disruption/breakdown with a large increase in barrier permeability and intense activation of microglial cells leading to drastic changes in morphologic phenotype, metabolic, and functional states (Schenk and de Vries, 2016; Sweeney et al., 2019; Wolf et al., 2017; Sierra et al., 2019). In contrast, considerably fewer studies were available on hypertension. Biancardi et al. (2014) reported BBB disruption within autonomic areas with access of fluorescent-labeled plasma Ang II into the brain parenchyma. The effects of Ang II and immune cells on BBB permeability within brain areas related to autonomic control were also analyzed, indicating the involvement of microglia as key cell targets (Biancardi et al., 2014; Setiadi et al., 2018). Microglia-mediated neuroinflammation was also suggested as a potential target for the treatment of hypertension (Wang et al., 2022). Studies from our laboratory in the chronic phase of spontaneous hypertension confirmed the permissive role of Ang II to drive BBB leakage within autonomic nuclei, showing that simultaneous intracerebroventricular infusion of Ang II abrogated the correction of BBB leakage induced by exercise training (Buttler et al., 2017). We also showed that the marked BBB dysfunction, allowing the access of plasma substances into the brain parenchyma, was caused by increased transcellular vesicle trafficking across the capillary endothelium (Fragas et al., 2021; Candido et al., 2023). In addition, strong positive correlations between vesicle trafficking and BBB leakage and between BBB permeability and hemodynamic/autonomic parameters were observed (Buttler et al., 2017; Fragas et al., 2021; Candido et al., 2023). The present set of data expanded our knowledge of the interaction between BBB dysfunction, microglia activation, and autonomic imbalance during the establishment of hypertension.

Our temporal analysis showed a small but significant increase in Ang II density within the PVN NTS and RVLM at the pre-hypertensive phase despite the presence of an intact BBB in the SHRs. Increased brain Ang II density preceding by 1–2 weeks the access of plasma Ang II through the BBB leakage (6th week on) suggests a local formation of the peptide. Previous articles on adult rat brains have already shown angiotensinogen synthesis by astrocytes as well as locally synthesized Ang II and higher renin–angiotensin system activity in hypertensive than normotensive controls (Gregory et al., 1982; Ganten et al., 1983; Stornetta et al., 1988). The present data on brain Ang II immunofluorescence supports these previous observations, indicating that local Ang II synthesis also occurred in the pre-hypertensive SHRs. This initial Ang II increase (5th week) was followed by incipient BBB leakage (6th week), and the barrier permeability increased continuously (6 weeks to 12 weeks), adding leaked plasma to locally synthesized Ang II. From 8 weeks on, there was a parallel increase in both Ang II-microglia colocalization and intense morphologic phenotype changes in microglial cells, as ratified by the huge changes observed in soma size, microglial processes, and endpoints. It should be noted that although microglia morphology did not equate with function, several studies indicated that morphologic phenotype is closely related to their function (Fernández-Arjona et al., 2017; Mowry et al., 2021; Paolicelli et al., 2022). Indeed, profusely ramified homeostatic microglia are considered anti-inflammatory and neuroprotective, while activated microglia with enlarged soma, shorter and fewer branches, and reduced endpoints are shown to secrete pro-inflammatory cytokines, such as TNFα, IL-1β, and IL-6 (Fernández-Arjona et al., 2017; Li et al., 2020; Mowry et al., 2021; Paolicelli et al., 2022). Interestingly, the association of intense BBB leakage, high Ang II availability, and altered microglia morphology (indicative of activated microglia) occurred simultaneously with hemodynamic (BP elevation; HR reduction) and autonomic responses (elevation of sympathetic vasomotor activity and pressure variability), suggesting that Ang II-activated microglia contribute to sympathoexcitation and autonomic imbalance during the transition from pre-to hypertensive phase in SHRs. Despite the high Ang II density within the NTS, its colocalization with microglial cells was much less than that exhibited by the PVN and RVLM. Therefore, Ang II-microglia association, a strong stimulus to potentiate the sympathetic activity (Shen et al., 2015; Li et al., 2020; Yu et al., 2022; Wang et al., 2022), is highly active within the PVN and RVLM, the main nuclei sending direct efferent projections to the sympathetic preganglionic neurons. Within the non-autonomic brain areas, increased Ang II density also preceded BBB dysfunction and microglial activation; Ang II-microglia colocalization and structural changes in microglial cells were also observed. However, these responses within sensory and motor integrative areas were lower than those exhibited by the PVN, NTS, and RVLM, indicating a clear predominance of hypertension-induced effects on autonomic areas.

It is well known that Ang II, via AT1 receptors, is an effective stimulus to activate both neurons and microglial cells, thus elevating the sympathetic activity, the pro-inflammatory profile, and the blood pressure (Biancardi et al., 2016; Forrester et al., 2018; Labandeira-Garcia et al., 2017; Li et al., 2020; Wang et al., 2022). Indeed, Ang II is a key factor in the establishment of hypertension, a low-grade inflammatory disease that affects over a quarter of the adult population in developed countries (Su et al., 2021). By treating 13-week-old SHRs with losartan or hydralazine and by injecting fluorescently labeled Ang II intravascularly, Biancardi et al. (2014) reported the essential role of Ang II to cross the disrupted BBB and colocalize with neurons and microglia within critical brain regions known to generate the autonomic drive. Several studies reported that Ang II, in addition to its direct effect on neurons increasing local inflammation and oxidative stress, also activated microglia, further increasing the expression of both pro-inflammatory cytokines and reactive oxygen species in hypertensive subjects (Cui et al., 2019; Kerkhofs et al., 2020; Mowry et al., 2021; Wang et al., 2022). Kerkhofs et al. (2020) showed that pharmacological depletion of microglia did not affect the increased BBB leakage and the blood pressure elevation in Ang II-infused mice; this observation supports our suggestion that Ang II, not microglia activation, is the key factor in triggering hypertension-induced responses. However, activated microglia, by augmenting local production of pro-inflammatory cytokines and increasing the oxidative stress (Cui et al., 2019), potentiated the BBB leakage, facilitating the sympathetic activation, autonomic imbalance, and blood pressure elevation observed in the present study. High oxidative stress and increased pro-inflammatory cytokine secretion were observed within the PVN of 12-week-old SHRs (Masson et al., 2014). Moreover, the comparison of SHR effects (increased Ang II availability preceding both BBB dysfunction and microglia structural changes and being followed by hemodynamic/autonomic responses) with those observed in age-matched normotensive controls (absence of these responses) supports our proposal that Ang II is the main stimulus to trigger BBB–microglia interplay not only in the chronic phase but also in the pre-hypertensive phase.

Our data on sequential morphological microglial changes within autonomic and non-autonomic areas revealed that the transition from homeostatic-surveilling to disease-associate condition during the establishment of hypertension involves altered microglial states with slight differences from one area to another. Consistent with these observations, Paolicelli et al. (2022) reported that microglia are the most dynamic cells of the mature brain, are modulated by local signals, and differ in morphology and functional specialization among central nervous system areas, according to their pathophysiological state. We also detected that except for a slight reduction in the RVLM (−15% on average), there was no change in microglial cell number within autonomic and non-autonomic areas during the development of hypertension. In contrast to inflammatory and neurodegenerative diseases that involve intense microglial proliferation (Wolf et al., 2017), hypertension, a low-grade inflammatory disease, did not change the number of microglial cells. In accordance, our data showed that during the establishment of spontaneous hypertension, microglia did not proliferate but changed their morphologic phenotype from highly ramified cells in a homeostatic condition of the pre-hypertensive SHRs to enlarged somas, shorter processes, and fewer endpoints in the chronic phase, indicative of the secretory phenotype. To confirm the structure–function relationship of microglial cells during the development of neurogenic hypertension, future studies should quantify the time-course changes of anti- and pro-inflammatory cytokines secretion during the establishment of hypertension.

A caveat of this study is that experiments were only made on male rats. However, previous studies on BBB permeability and transcytosis changes in embryonic brains and pups found no sex-based differences (Ben-Zvi et al., 2014; Andreone et al., 2017). In immature male and female rodent brains, significant differences in microglia number and morphology were found in the preoptic area, hippocampus, parietal cortex, and amygdala, but these differences do not map to a clear microglial phenotype, as observed in surveillant or injured adult brains (Lenz and McCarthy, 2015). Moreover, considering that the renin–angiotensin system is not gender-specific and that neurogenic hypertension accompanied by autonomic imbalance occurs in both male and female SHRs, we believe that similar BBB disruption and microglia morphological changes may also characterize the transition from the pre-to hypertensive phase in female rats.

In conclusion, our data indicated that increased brain Ang II density within autonomic and non-autonomic areas is the first detectable stimulus to drive coordinated changes in local BBB permeability and microglial responsiveness during the transition from the pre-hypertensive to the chronic phase of spontaneous hypertension. BBB dysfunction, by allowing the entrance of plasma Ang II into the brain parenchyma, activates a vicious cycle by which increased brain Ang II availability further potentiates both barrier permeability and microglial activation during the development of hypertension. The maintenance of disease-associated conditions continuously increases sympathetic activity and pressure variability, determining the displacement of blood pressure toward elevated levels in the chronic phase of hypertension.

## Data Availability

The original contributions presented in the study are included in the article/Supplementary Material; further inquiries can be directed to the corresponding author.
